# Real benefits of ultrasound evaluation of hand and foot synovitis for better characterisation of the disease activity in rheumatoid arthritis

**DOI:** 10.1007/s00330-019-06187-8

**Published:** 2019-04-26

**Authors:** Coziana Ciurtin, Alexis Jones, Geraint Brown, Fang En Sin, Charles Raine, Jessica Manson, Ian Giles

**Affiliations:** Department of Rheumatology, University College London Hospitals NHS Trust, 250 Euston Road, London, NW1 2PG UK

**Keywords:** Doppler ultrasound, Rheumatoid arthritis, Synovitis, Outcome measures

## Abstract

**Objectives:**

Optimal management of rheumatoid arthritis (RA) depends on accurate evaluation of disease activity. Foot synovitis is not included in the most used RA outcome measure (DAS-28 score). The aim of this study was to investigate how musculoskeletal ultrasound (MSK-US) examination of hand and feet correlate with the disease activity score (DAS-28 score). We also explored whether performing MSK-US assessments of hands alone compared with hands and feet underestimates the disease activity in RA.

**Methods:**

This is a real-life cross-sectional study of 101 patients (51 with RA and 50 with other musculoskeletal conditions) with inflammatory small joint pain, who underwent MSK-US examination of hands and feet.

**Results:**

MSK-US-detected hand synovitis was found in 18/51 (35.3%) RA patients and 16/50 (32%) of those with other musculoskeletal conditions (*p* = 0.96), while foot synovitis was detected in 18/51 (35.3%) and 12/50 (24%) patients, respectively (*p* = 0.78). DAS-28 did not correlate with any of the US outcome measures in patients with RA. Six out of 13 (46.1%) RA patients in remission, 7/14 (50%) with low disease activity and 18/32 (56.2%) with moderate disease activity (according to DAS-28 definition) had active synovitis as assessed by the MSK-US examination of their hands and feet. MSK-US-detected synovitis led to treatment escalation in 26/51 (51%) RA patients.

**Conclusion:**

This study emphasises that MSK-US examination of hands and feet has led to optimised management of the majority of RA patients, which would have not been possible otherwise, because of the lack of correlation between DAS-28 assessment and MSK-US outcomes.

**Key Points:**

*• The most used disease activity score in rheumatoid arthritis (DAS-28) did not correlate with US outcome measures derived from hands and feet examination.*

*• DAS-28 did not differentiate between RA patients with subclinical active synovitis* versus *well-controlled disease on US.*

*• As a result of US examination of the hands and feet, 51% RA patients had their immunosuppressive treatment optimised.*

**Electronic supplementary material:**

The online version of this article (10.1007/s00330-019-06187-8) contains supplementary material, which is available to authorized users.

## Introduction

Rheumatoid arthritis (RA) is a systemic autoimmune disease characterised by polyarticular synovial inflammation, leading to bone destruction and deformity. Management of RA incorporates a treat-to-target approach with the aim of achieving clinical remission as soon as possible [1]. Numerous studies have demonstrated that early treatment improves clinical outcomes and reduces radiographic progression [2].

## Background and rationale

The “treat to target” approach is guided by disease activity scores, which were initially used in clinical trials before being implemented in clinical practice as guidance for treatment optimisation for better disease control [3, 4]. The most frequently used outcome measure is the disease activity score DAS-28; a composite score of the number of tender and swollen joints out of a 28-joint assessment, a patient-derived global visual analogue score (VAS) and either erythrocyte sedimentation rate (ESR) or C-reactive protein (CRP) [5]. There are a number of criticisms of this score, notably the subjectivity of the joint assessment, the poor specificity of global VAS which can be affected by a number of factors other than RA and the lack of appreciation of foot arthritis [6]. Advances in musculoskeletal ultrasound (MSK-US) technology and the increasing number of rheumatologists trained in this skill have increased the availability of ultrasound (US) and its use as an objective measure of inflammation in inflammatory arthritis. Previous studies by ourselves and others found that a variable proportion (26.8–50%) of RA patients in DAS-28 remission had active synovitis in at least one joint on hand US [7, 8], while others questioned the reliability of DAS-28 assessment. A study published in an abstract form only reported US-detected synovitis in 42–82% hand joints and 18–27% foot joints in RA patients classified as being in remission using various disease activity scores [9]. Subclinical synovitis was also found using MSK-US at the wrist (36.5%) and feet (33.7%) level in early psoriatic arthritis [10].

## Aim

There is an unmet clinical need to explore the real-life benefits of MSK-US examination of the hands and feet for diagnosis and management of patients with RA and other inflammatory arthritides.

The aim of this study was to investigate in a single-centre, cross-sectional study the impact of MSK-US on the “day to day” diagnosis and management of RA patients. Specifically, this study examined how MSK-US outcome measures correlate with the validated DAS-28 score, and whether performing MSK-US assessments of the hands alone compared with MSK-US examination of the hands and feet underestimates the disease activity in RA patients.

## Materials and methods

### Study design and inclusion criteria

This is a single-centre, cross-sectional study, which evaluated consecutive patients with various rheumatic conditions referred to a dedicated MSK-US clinics at the University College London Hospital (UCLH), London, UK, between October 2015 and April 2017. All the patients included in this study had symptoms suggestive of inflammatory joint pain in their hands and feet, irrespective of their underlying diagnosis, and were referred by their consultant clinician to have an MSK-US scan of their small joints to aid identification of joint inflammation for diagnostic and prognostic purposes. All clinicians are aware of the existence of our service and refer patients based on clinical indication (there was no obvious selection bias).

The research methods used in this study were clinical examination, blood tests for serological markers of inflammation, patient-reported outcomes, a validated disease activity score (DAS-28) and US examination of the hands and feet as per RA protocols published before [11] (see details below).

### Study population

**Fig. 1:**
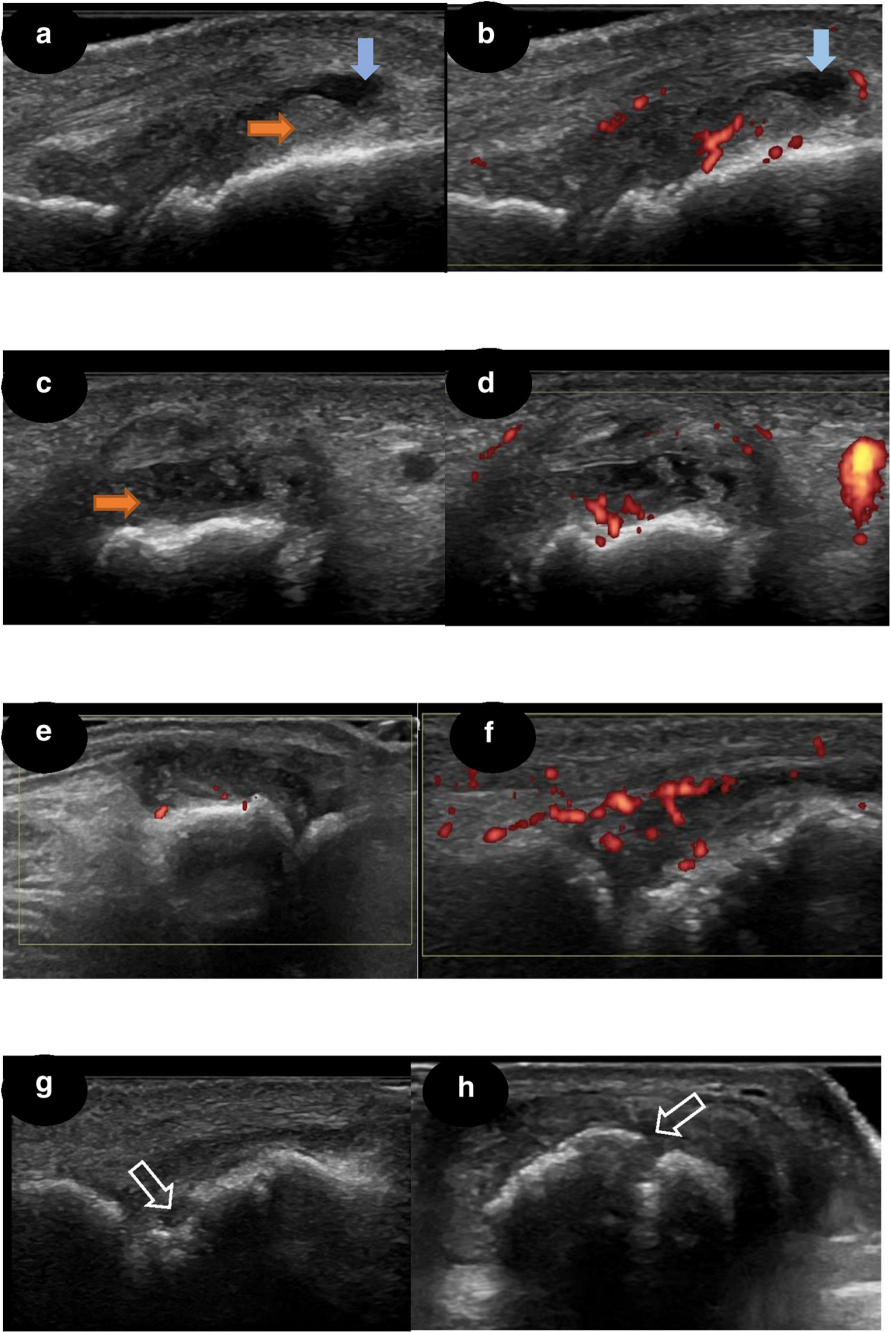
Examples of US grading. **a**–**d** MCP joint with effusion, SH grade 3 and PD grade 2, longitudinal and transversal views. **e** PIP joint with SH grade 3 and PD grade 1. **f** PIP joint with SH grade 2 and PD grade 3. **g**, **h** PIP joint with erosion, longitudinal and transversal views; white arrow, erosion; orange arrow, SH; blue arrow, joint effusion; SH, synovial hypertrophy; PD, power Doppler signal

Of the 503 consecutive patients referred to the rheumatologist-led MSK-US service at the UCLH between October 2015 and April 2017, 111 patients were referred for MSK-US examination of their hands and feet to evaluate clinical suspicion of active inflammatory joint disease. A final total of 101 patients were included in the study (79 females, 22 males), while 10 patients were excluded owing to incomplete data collection. The mean age of the total cohort was 43.5 years. Of these, 51 patients had a confirmed (pre-existing) or received a diagnosis of RA based on the modified American College of Rheumatology (ACR) 1987 criteria [12] or the ACR/European League Against Rheumatism (EULAR) 2010 classification criteria [13]. The remaining 50 patients with joint pains in their hands and feet were used in this study as the non-rheumatoid control group. A total of 54% of these patients were previously diagnosed with other musculoskeletal conditions (including seronegative inflammatory arthritis, gout, fibromyalgia, osteoarthritis and SLE), while 46% had no identifiable cause for their arthralgia following clinical assessments, laboratory investigations and MSK-US scan of the hands and feet (see Figure 1, supplementary information).

### Disease assessment

We collected information about disease duration (in months), clinical joint examination findings including hand tender joint count (TJC) and hand swollen joint count (SJC) out of 28 joints included in the DAS-28 score, in addition to clinical assessment of 10 metatarsophalangeal (MTPs) joints. Patient-reported global assessment score (GVAS) and pain scores (on a scale 1 to 10) were also recorded. Additional data about the high sensitivity C-reactive protein (hsCRP), ESR, presence of rheumatoid factor (RF), anti-citrullinated cyclic peptides antibodies (ACPA) and anti-nuclear antibodies (ANA) was also available at the time of the scan.

### DAS-28 score definitions of disease activity states

DAS-28 score is a composite outcome measure of disease activity in RA comprising a 28 tender joint count (range 0–28), a 28 swollen joint count (range 0–28), ESR and an optional general health assessment on a visual analogue scale (range 0–100) [14]. A DAS-28 value > 5.1 corresponds to a high disease activity; a DAS-28 value between 3.2 and 5.1 corresponds to a moderate disease activity; a DAS-28 value between 2.6 and 3.2 corresponds to a low disease activity, while a DAS28 value < 2.6 corresponds to remission [14, 15].

### MSK-US examination

An established protocol of MSK-US examination of the hands comprising 22-joint assessments (dorsal longitudinal and transverse views of wrists, including intercarpal, radial and ulnar views; metacarpophalangeal (MCPs) and proximal interphalangeal (PIPs) joints) and 10 metatarsophalangeal (MTP) joints was used [11]. The presence of active joint inflammation was defined as power Doppler (PD) signal within a region of grey scale (GS) synovitis, which was graded 1–3 (grade 1, up to three single Doppler spots or up to one confluent spot and two single spots or up to two confluent spots; grade 2, greater than grade 1 but < 50% Doppler signals in the total GS background; grade 3, greater than grade 2 (> 50% of the background GS)). GS synovitis was graded 1–3 according to the severity of synovial hypertrophy (SH): grade 1, SH with or without effusion up to level of horizontal line connecting bone surfaces; grade 2, SH with or without effusion extending beyond joint line but with upper surface convex (curved downwards) or hypertrophy extending beyond the joint line but with upper surface flat; grade 3, SH with or without effusion extending beyond the joint line but with upper surface flat or convex (curved downwards); and joint effusion graded as present/absent, as per the Outcome Measures in Rheumatoid Arthritis Clinical Trials (OMERACT) US definitions developed for RA [16]. Erosions were defined as an intra-articular discontinuity of the bone surface that is visible in two perpendicular planes [17] (Fig. 1). In addition, the presence of active inflammation (PD signal) within the MTP1 bursa (bunion) was not counted towards the MSK-US feet PD scores.

MSK-US examination was performed by two rheumatologists, one with 7-year experience (CC) and another one with 12-month experience in MSK MSK-US (AJ). For every patient, a consensus regarding the scoring of every joint was obtained.

MSK-US examination was performed using a Logiq S8 US machine (GE Medical Systems Ultrasound and Primary Care Diagnostics,), equipped with a multi-frequency linear matrix array transducer (8–15 MHz). B-mode and PD (machine settings) were optimised for all MSK-US examinations as per OMERACT recommendations [16]. We used the PD factory setting for superficial musculoskeletal assessment which was adjusted for increased Doppler sensitivity by decreasing the wall filter and pulse repetition frequency and adjusting the Doppler gain to the level just below random noise. The setting was saved and used for every patient US examination, as per previously published recommendations [18]. The GS synovitis score and the PD score were calculated as previously described [16]. Total GS and PD scores per patient were calculated as the sum of individual GS and PD joint scores. The erosion score was calculated as the total number of erosions per patient (as all the joints have been scored in a binary manner: 1, present; 0, absent). Data on active inflammation or chronic inflammatory changes affecting the tendons overlying the abovementioned joints was also collected, but the analysis of tendon pathology was beyond the scope of this paper.

The duration of MSK-US examination, including 32 joints (hands and feet), including scoring of MSK-US parameters took approximately 30 min per patient.

### Treatment optimisation

All patients with active synovitis in their joints had treatment optimisation following the MSK-US assessment, including systemic therapy (escalation of disease-modifying anti-rheumatic drugs—DMARD therapy, addition or increase in the oral/intramuscular steroids) or local therapy (US-guided intra-articular injections).

### Statistical analysis

All data was transferred and collated from paper questionnaires to a Microsoft Excel spreadsheet. IBM SPSS Statistics 22 (IBM 2013) was used for further analysis and statistical tests. Descriptive statistics tests were employed to characterise the patient population further, using mean and standard deviations (SD) and median with inter-quartile ranges (IQR) depending on the data distribution. A *p* value of < 0.05 was considered significant. Spearman’s correlation coefficients were used to correlate clinical and MSK-US parameters, while Pearson’s *R* or phi coefficient was used to assess the correlation between dichotomous variables.

## Results

### Patient characteristics and comparison between RA and control groups

Demographic, serologic, clinical features and MSK-US findings of the patient groups are summarised in Table 1. Of the 51 patients with RA, 44/51 (86%) of patients were women, 37/51 (72.5%) were RF-positive and 32/51 (62.7%) were ACPA-positive. In the RA group, 66.7% of patients were on DMARDs (statistically significant difference from the non-RA control group *p* < 0.0001), while only 3/51 (5.85%) were on biologic therapy at the time of the scan. Aside from these expected serologic and treatment differences, there were no other significant differences in age, symptom duration, TJC, SJC, CRP, ESR and GVAS between the RA and non-rheumatoid control group. The average DAS-28 score in the RA group was 3.64 ± 1.55. There was no significant difference, however, between the total number of swollen and tender joints at the time of MSK-US examination between the two patient groups, which explains the clinician decision to refer all these patients for additional MSK-US evaluation of their small joints.

**Table 1:** Comparison between the patient groups

	RA	Non-rheumatoid control group	*p* value
Number (F:M)	51 (44:7)	50 (35:15)	0.057
Age (mean years +/−SD)	45.3 (± 14.9)	42 (± 12.34)	0.85
Symptom duration, (mean years +/−SD)	5.28 ± 3.67	5.32 ± 4.21	0.62
Patients on steroids at the time of the scan *Includes IM/IV within 3 months*	7 (13.7)	1 (2.0)	0.24
Patients on DMARDs at the time of the scan n (%)	34 (66.7)	8 (16.0)	*< 0.0001*
Patients on biologic treatment at the time of the scan n (%)	3 (5.8)	2 (4.0)	0.238
CRP (mean ± SD)	3.75 (± 4.99)	4.37 (± 6.25)	0.32
ESR (mean ± SD)	14.56 (± 17.6)	13.3 (± 16.5)	0.58
Hand SJC (median, IQR)	0 (0–5)	3 (0–13)	0.18
Hand TJC (median, IQR)	3 (0–28)	3 (0–28)	0.31
% patients with hand symptoms	100	100	N/A
Feet SJC (median, IQR)	1 (0–5)	2 (0–10)	0.19
Feet TJC (median, IQR)	3 (2–5)	3 (1–10)	0.67
% patients with feet symptoms	100	100	N/A
Pain VAS mean +/− SD	51.8	49.8	0.4
Rheumatoid factor (%)	37 (72.5)	8 (16)	*0.0002*
ACPA (%)	32 (62.7)	4 (8)	*0.0002*

*P* < 0.05 was considered significant

*ACPA*, anti-cyclic citrullinated peptide antibodies; *CRP*, C-reactive protein; *ESR*, erythrocyte sedimentation rate; *IQR*, inter-quartile range; *SD*, standard deviation; *SJC*, swollen joint count; *TJC*, tender joint count; *VAS*, visual analogue scale

### MSK-US-detected synovitis in RA versus non-rheumatoid patient groups

To evaluate whether there were significant differences between the MSK-US parameters of the hand and/or foot exam in the RA group compared with the non-rheumatoid control group, the PD scores (assessing for the evidence of active synovitis), GS score (assessing for the presence of chronic inflammatory joint changes) and erosion scores (assessing joint damage) were compared between these two patient groups. The proportion of patients with active hand and foot synovitis (PD ≥ 1) on MSK-US was similar between the two patient groups (18/51 (35.3%) RA patients versus 16/50 (32%) in the non-rheumatoid control group, *p* = 0.96, while 18/51 (35.3%) versus 12/50 (24%) patients respectively have been found with foot synovitis, *p* = 0.78).

As data on MSK-US joint abnormalities in patients with other inflammatory arthritides or without inflammatory pathology (such as fibromyalgia or pain associated with hypermobility) are generally lacking, we aimed to assess the differences in the burden of hand and foot synovitis between in the two patient groups. Therefore, we compared the MSK-US scores for hands (wrist, MCP and PIP joints; assessing 22 joints) and the MSK-US scores for feet (assessing 10 MTP joints) separately.

There was a statistically significant difference in PD, GS and erosion scores in the hands but not in the feet between patients with RA and those in the non-rheumatoid control group (Table 2). This is unsurprising, as hand joint involvement is considered the hallmark of RA. Notably, 7 of 50 patients in the control group had a diagnosis of fibromyalgia and no detectable PD or other significant abnormalities on MSK-US examination of either hands or feet.

**Table 2:** Comparison between the US findings between the RA and non-rheumatoid control group

	RA	Non-RA	*p* value
MSK-US findings (22 hand joint examination) RA vs. non-RA			
Mean PD score (SD)	1.82 (± 4.39)	0.86 (± 3.29)	*0.0001*
Mean GS score (SD)	7 (± 10)	2.98 (± 7.80)	*0.003*
Mean erosion score (SD)	3.61 (± 6.11)	0.86 (± 2.72)	*0.003*
Number of patients with PD ≥ 1 in their hands	18/51 (35.3%)	16/50 (32%)	0.96
MSK-US findings (10 MTP joint examination) RA vs non-RA			
Mean PD score (SD)	0.76 (± 1.35)	0.36 (± 1.40)	0.93
Mean GS score (SD)	6.18 (± 7.39)	3.88 (± 5.24)	0.58
Mean erosions score (SD)	1.39 (± 1.5)	0.38 (± 0.85)	0.79
Number of patients with PD ≥ 1 in their feet	18/51 (35.3%)	12/50 (24%)	0.78

*P* < 0.05 was considered significant

*GS*, grey scale; *MSK-US*, musculoskeletal ultrasound; *PD ≥ 1*, power Doppler signal ≥ 1; *RA*, rheumatoid arthritis; *SD*, standard deviation

### DAS-28 did not correlate with MSK-US outcome measures in RA patients and did not help differentiate between patients with subclinical synovitis versus well-controlled disease

There was no statistically significant correlation between any of the MSK-US scores and the DAS-28 score in the RA patient group (Table 3). However, statistically significant correlations were found between GS score and the ESR component of DAS-28 (*R* = 0.41, *p* < 0.05).

**Table 3:** Correlation table (*R*^2^) for disease activity outcomes in RA patients

	SJC	TJC	PD score	GS score	Erosions score	ESR	CRP	VAS
DAS-28	0.38*	0.59	0.29	0.18	0.21	0.61	0.20	0.70
SJC	1.0	0.18	0.61	0.58	0.37	0.24	0.12	0.10
TJC		1.0	0.05	0.03	0.15	0.29	0.06	0.50*
PD score			1.0	0.71	0.08	0.11	0.09	0.15
GS score				1.0	0.11	0.41*	0.05	0.01
Erosions					1.0	0.16	0.02	0.01
ESR						1.0	0.02	0.27
CRP							1.0	0.003
VAS								

*CRP*, C-reactive protein; *ESR*, erythrocyte sedimentation rate; *DAS-28*, disease activity score assessing 28 joints; *GS*, grey scale; *PD*, power Doppler; *SJC*, swollen joint count; *TJC*, tender joint count

*Denotes *p* value < 0.005

Statistical significance was also found between components of the DAS-28 score, as expected, specifically between DAS-28 and SJC (*R* = 0.38, *p* < 0.05), and between TJC and VAS (*R* = 0.5, *p* < 0.05).

**Fig. 2:**
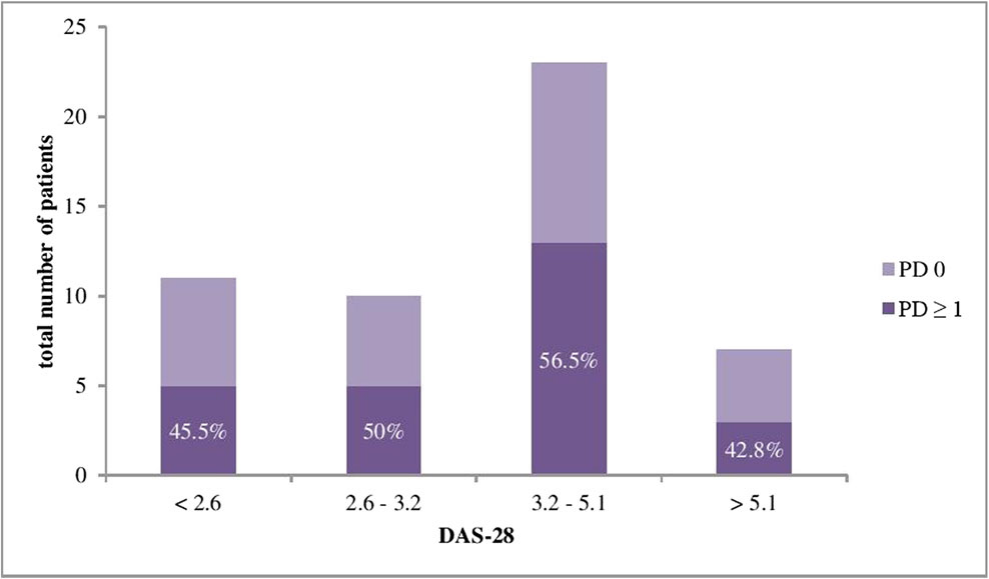
Proportion of patients with US-detected active synovitis either in hands or feet, or both, stratified based on DAS-28 scores. DAS-28, disease activity score assessing 28 joints; PD, power Doppler score

As MSK-US examination is recognised as being more sensitive than clinical examination, this study assessed the proportion of RA patients with active synovitis (defined as a PD ≥ 1), either in their hands or feet (or both) within each disease activity group, as defined by the DAS-28 scores. Six out of 13 patients (46.1%) of RA patients classified as being in remission and 5/12 (41.7%) of patients with severe disease activity had active synovitis in at least one joint (PD ≥ 1). The highest proportion of patients with MSK-US-detected active synovitis was found in the low and moderate disease activity RA groups: 7/14 (50%) and 18/32 (56.2%), respectively. Figure 2 details the number of RA patients with or without MSK-US-detected active synovitis within each DAS-28 disease activity interval.

### MSK-US examination of feet (in addition to hands) improved the sensitivity of MSK-US for diagnosis of active synovitis in RA patients in remission, and with low and moderate disease activity

In this study, a significant proportion of the RA patients had evidence of foot arthritis on MSK-US, despite DAS-28 score suggesting remission (4/11, 36.4%) and despite having no active inflammatory changes in their hands (2/11, 18%).

**Fig. 3:**
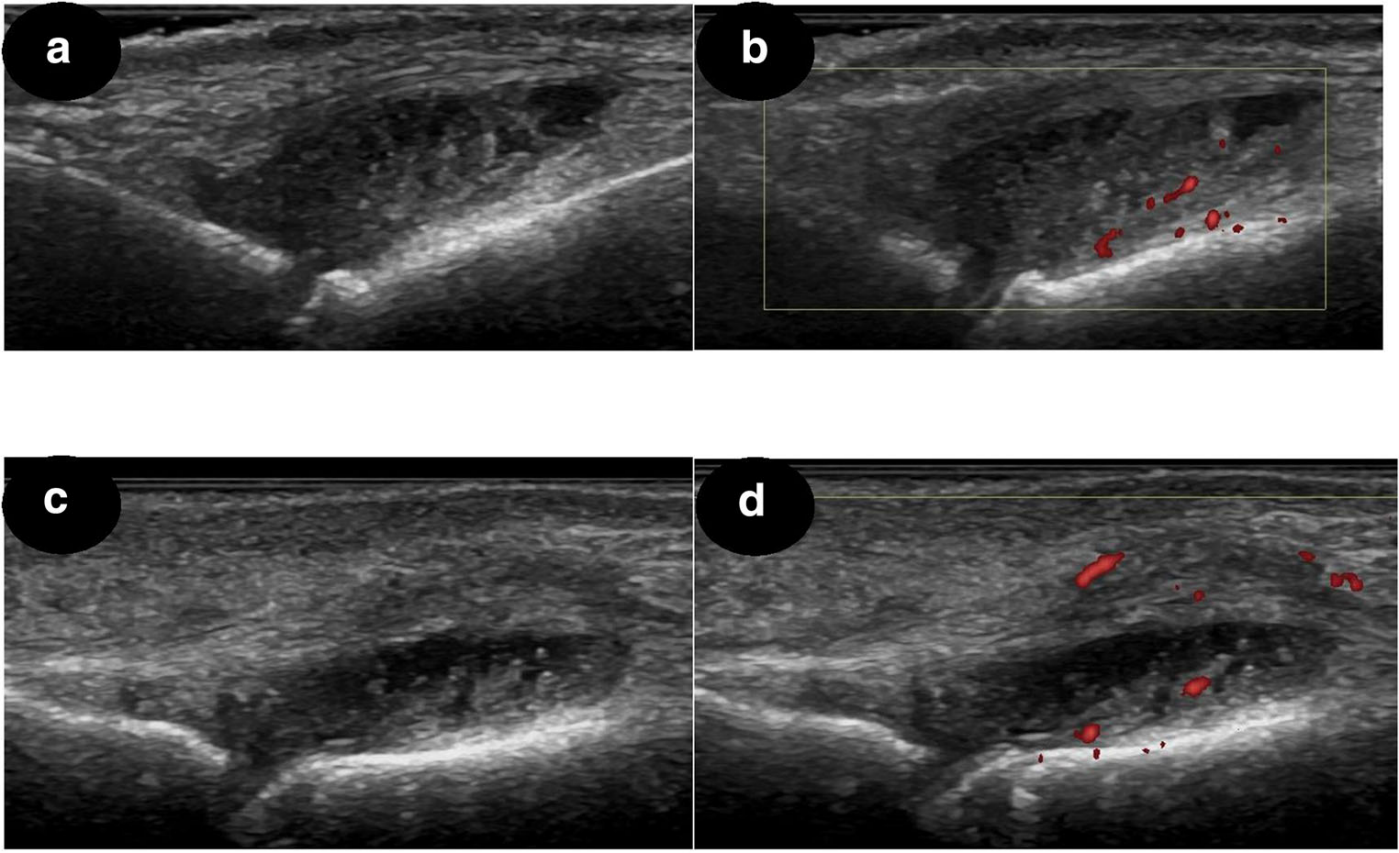
MSK-US findings in an RA patient in remission (DAS-28 = 2.1). **a**, **b** MTP joint with SH grade 3 and PD grade 1. **c**, **d** PIP joint with SH grade 3 and PD grade 1

Given that one of the main purposes of this study was to examine the utility of an extended MSK-US protocol (hands and feet) for the evaluation of disease activity in RA, we compared the sensitivity of hand MSK-US protocol to one assessing both hands and feet in RA patients stratified according to their DAS-28 scores (Table 4**)**. The results showed that by simplifying the MSK-US examination to include only hands, a significant proportion of RA patients in DAS-28 remission (60%), with low disease activity (20%) and with moderate disease activity (31.8%), were wrongly diagnosed as having well-controlled disease. In the group of RA patients with DAS-28 > 5.1, the same proportion of patients were diagnosed as having active synovitis on MSK-US, irrespective of having their feet scanned in addition to hands or not. Figure 3 details the MSK-US findings of one RA patient classified as being in clinical remission based on DAS-28 score (DAS-28 = 2.1), but who had active synovitis in two joints (one MTP and one PIP joint). MSK-US-detected hand synovitis did not correlate with foot synovitis in RA patients.

**Table 4:** Sensitivity of MSK-US hands versus hands and feet in detecting active synovitis in RA patients stratified based on their DAS-28 scores

DAS-28	Patients with PD ≥ 1 in their hands (irrespective of having it or not in their feet)	Patients with PD ≥ 1 in their feet (irrespective of having it or not in their hands)	Patients with PD ≥ 1 in both hands and feet	Sensitivity of MSK-US hands vs. hands and feet (%)
< 2.6	2	4	5	40
2.6–3.2	4	3	5	80
3.2–5.1	9	9	13	69.2
> 5.1	3	2	3	100
Total patients	18	18	26	

*DAS-28*, disease activity score assessing 28 joints; *MSK-US*, musculoskeletal ultrasound; *PD*, power Doppler

In this study, there was no correlation between MSK-US outcome measures in hands and MSK-US outcome measures in feet in RA patients (Table 1 and Figure 2 supplementary information).

Although RA patients had more active synovitis (mean PD score 1.82 versus 0.76, *p* = 0.04), and more erosions (mean erosion score 3.61 versus 1.39, *p* = 0.021) in their hands compared to their feet, there was no significant difference between the mean GS score between hands and feet (7 versus 6.18, *p* = 0.76). In addition, the same number of RA patients had active synovitis in their hands as in their feet (*n* = 18, 35.3%) (Figs. 3 and 4, supplementary information), although 10/51 patients (19.6%) were found to have active foot arthritis despite the lack of active synovitis in their hands (PD = 0). These patients would have been wrongly diagnosed as being in remission if only their hands were examined. In addition, all the RA patients with active synovitis in their feet alone were classified by DAS-28 as being in remission or having low disease activity, as suggested by the lack of correlation between the MSK-US and DAS-28 outcome measures.

### Added role of MSK-US examination of small joints for management optimisation

Overall, the MSK-US examination of the hands and feet assisted the diagnosis in 14/51 (27.5%) of RA patients (new onset RA) as it confirmed the presence of active synovitis which has prompted initiation of specific RA treatment. The remaining 37/51 (72.5%) patients, previously diagnosed and treated for RA, had treatment optimisation guided by the results of the MSK-US scan. 12/51 RA patients (23.5%) have been prescribed escalation of conventional/biologic therapy or intra-articular or oral steroids, and the remaining 25/51 patients (49%) had been referred for supportive treatment for better pain management. Thus, overall, 51% (26/51) of RA patients (newly diagnosed or with an established disease) have been recommended changes in their immunosuppressive treatment as a result of MSK-US examination of hands and feet.

In the non-rheumatoid group, the MSK-US examination of small joints has led to the escalation of immunosuppressive therapy in a proportion of 36% (18/50) patients.

## Discussion

MSK-US examination of hands and feet has found a similar proportion of patients with active synovitis in both RA and non-rheumatoid patient groups, which is not surprising considering the patient selection criteria. The key result of this study is the added value of an MSK-US examination protocol including hands and feet for optimal diagnosis and management of RA patients with small joint symptoms. The results of this study have practical implications particularly that by using DAS-28 assessment alone to guide therapeutic decisions in RA, clinicians are potentially missing a significant proportion of patients with active disease. We also identified for the first time the discrepancy between the MSK-US-detected synovitis in hands versus feet in RA patients, emphasising that active inflammation in hand joints is not a predictor for active synovitis affecting the feet and vice versa, even if RA patients had symptoms in both their hands and feet. Furthermore, we found added value of an MSK-US protocol including feet examination for more accurate assessment of RA patients who have hand and feet pain, despite being classified by DAS-28 as being in remission or having low disease activity.

## Interpretation

MSK-US detection of active synovitis has practical implications to improve the management of RA and non-rheumatoid patients with hand and foot pain. The unrecognised burden of synovitis would explain why RA patients with seemingly controlled disease as defined by DAS-28 continue to develop bone damage and erosions [19]. Various studies examined the superiority of different types of imaging techniques for assessment of synovitis associated with RA [20–22] and the sensitivity of various MSK-US protocols for examination in RA [23]. Damajanov et al [24] tested a composite US DAS index, combining the values of PD, GS, laboratory and clinical variables, and investigated the validity and reliability of this test compared with the DAS-28 in RA patients. Brown et al [25] were the first to show that MSK-US-detected subclinical synovitis can lead to radiographic progression, even in clinical remission, while another study showed that MSK-US-detected synovitis was a better predictor than clinical examination for subsequent structural deterioration in patients with RA [26]. Despite previous studies demonstrating a good correlation between hand or hand and wrist MSK-US outcome measures, clinical examination and DAS-28 assessment [27, 28], we did not find a correlation between DAS-28 and any of the MSK-US scores derived from a protocol examining hands and feet. This suggests a clear disparity between the two types of outcome measures (DAS-28- and MSK-US-derived outcomes), when feet are also included in the MSK-US examination protocol.

Foot synovitis has been previously shown to be associated with adverse radiographic and functional outcomes and was underestimated by disease activity scores [29]. In one study, foot synovitis was found in 25% of patients with early RA who have otherwise achieved remission on the basis of standard disease activity measures [30], while in asymptomatic RA patients, active synovitis at the foot level was found in 5.77% of patients [31].

Failure to identify and thus treat appropriately this “subclinical” synovitis can lead to progressive joint damage [29]. Although we could not address this issue in our cross-sectional study, the radiographic burden of foot synovitis has been assessed longitudinally in RA. In particular, annual radiographs of hands and feet have shown increased erosive changes and joint space narrowing at baseline and cumulatively over a 6-year period in feet compared with hands in patients with early RA at study onset receiving DMARDs [32].

## Limitations

The main limitation of this study is related to the lack of strict inclusion and exclusion criteria for patients, who have been referred to have an US scan based on clinical indication and as decided by their rheumatologist. Although all patients referred to our MSK-US service were scrutinised for inclusion in this study, the authors cannot account for the inclusion bias related to their clinician preference to refer them for an US scan or not. In addition, this study was a single-centre study in a tertiary rheumatology centre, and the US examination was performed by two ultrasonographers with different degrees of experience (although a consensus was obtained for every patient). Therefore, the patient population and results cannot be generalised. Another potential limitation is that the involvement of large joints and tendons in RA and other inflammatory musculoskeletal conditions was not included in the MSK-US protocol used in this study. The authors are fully aware that our findings may not be directly applicable to RA patients with active synovitis that is clearly detectable on clinical assessment, as they are not usually referred for MSK-US assessment.

In conclusion, our study emphasises the significant and under-appreciated burden of foot arthritis in RA and the limitations of a simplified MSK-US protocol (assessing only 22 hand joints) compared with a protocol that assesses hands and feet (assessing 32 joints). Further research into improved long-term outcome of RA patients using this extended MSK-US small joint protocol versus clinical examination and DAS-28 assessment is needed to assess its potential clinical utility in routine practice.

## Electronic supplementary material

ESM 1 (DOCX 26 kb)

## Abbreviations

ACPA: Anti-citrullinated cyclic peptide antibodies
ACR: American College of Rheumatology
ANA: Anti-nuclear antibodies
CRP: C-reactive protein
DMARDs: Disease-modifying anti-rheumatic drugs
ESR: Erythrocyte sedimentation rate
GS: Grey scale
MCP: Metacarpophalangeal
MSK-US: Musculoskeletal ultrasound
MTP: Metatarsophalangeal
PD: Power Doppler
PIP: Proximal interphalangeal
RA: Rheumatoid arthritis
RF: Rheumatoid factor
SJC: Swollen joint count
TJC: Tender joint count
VAS: Visual analogue score
