# Supplementary material for: Real benefits of ultrasound evaluation of hand and foot synovitis for better characterisation of the disease activity in rheumatoid arthritis
Source: Eur Radiol. 2019 Apr 26;29(11):6345–54. doi: 10.1007/s00330-019-06187-8 (PMC6795612; doi:10.1007/s00330-019-06187-8)
Supplement: Supplementary file 1 — (DOCX 26 kb) [file 330_2019_6187_MOESM1_ESM.docx]

**Supplementary information**

**Figure 1 supplementary information**: Non-rheumatoid control group

**Table 1 supplementary information:** Relationship between foot and hand US outcomes in RA patients

|  | **Hands (mean)** | **Feet (Mean)** | **P value** | **R value** |
| --- | --- | --- | --- | --- |
| **PD** | 1.82 | 0.76 | 0.59 | 0.07 |
| **Gray scale** | 7 | 6.18 | 0.59 | 0.07 |
| **Erosions** | 3.61 | 1.39 | 0.87 | 0.57 |

**Figure 2 supplementary information:** Correlation between hands versus feet PD scores in RA patients

**Figure 3 supplementary information:** active synovitis found on US of hands in RA patients stratified based on DAS-28 (n = 18)

**Figure 4 supplementary information:** active synovitis found on US of feet in RA patients stratified based on DAS-28 (n = 18)
